# Frontiers of Molecular Biology of Cancer

**DOI:** 10.3390/ijms242417187

**Published:** 2023-12-06

**Authors:** Stergios Boussios, Elisabet Sanchez, Matin Sheriff

**Affiliations:** 1 Department of Medical Oncology, Medway NHS Foundation Trust, Gillingham ME7 5NY, UK; elisabet.sanchez@nhs.net; 2 Faculty of Life Sciences & Medicine, School of Cancer & Pharmaceutical Sciences, King’s College London, Strand, London WC2R 2LS, UK; 3 Kent Medway Medical School, University of Kent, Canterbury CT2 7LX, UK; 4 AELIA Organization, 9th Km Thessaloniki—Thermi, 57001 Thessaloniki, Greece; 5 Department of Urology, Medway NHS Foundation Trust, Gillingham ME7 5NY, UK; matin.sheriff@nhs.net

Cancer is rooted in genetic background, with the expression of oncogenesis playing a pivotal role in the early stages of tumor formation. Acquired mutations in somatic cells primarily contribute to the development of most common cancers, while specific germline mutations are responsible for rare hereditary cancer syndromes. Within the realm of cancer-associated genes, oncogenes undergo activation, exhibiting phenotypic dominance, whereas tumor suppressor genes experience inactivation, displaying phenotypic recessiveness. The ongoing effort to improve our knowledge about molecular mechanisms involves defining pathways influencing cancer therapy. Technological advancements have made it possible to identify genes integral to cancer development and have significantly contributed to the growing success of precision medicine in oncology, with targeted therapies directed against tumors and components of the tumor microenvironment. This Special Issue, titled “Molecular Biology of Cancer—Implications for Diagnosis and Treatment”, comprises a total of eight contributions. These include five original articles and three reviews, offering fresh insights into cancer biology, molecular genetics, and innovative therapeutic approaches.

Breast cancer (BrC) predominantly affects women, particularly those who are postmenopausal. The incidence rates have shown an upward trend in recent years, impacting 2.26 million females in 2020, with an estimated 2.5 million new cases predicted by 2025 [1]. Zarychta et al.’s study provided a comprehensive examination of angiogenic parameters in BrC, both pre- and post-treatment, and investigated their correlation with specific treatment modalities (contribution 1). The study revealed that elevated pre-treatment levels of the soluble form of vascular endothelial growth factor receptor type 1 (sVEGFR1) and reduced pre-treatment levels of sVEGFR2 are associated with improved disease-free survival (DFS) outcomes. Furthermore, the research identifies significant connections between various BrC treatments, including surgery, radiotherapy, chemotherapy, and endocrine therapy, and alterations in angiogenic parameters. Notably, higher post-treatment levels of sVEGFR2 are linked to improved overall survival (OS), as indicated by Kaplan–Meier analysis. These findings highlight the potential significance of angiogenic parameters as markers for predicting treatment response and patient outcomes in BrC.

Al Quatami et al. employed in silico assays to explore the bidirectional impact of neutrophils on metastatic triple-negative BrC (contribution 2). Their analysis revealed that the majority of triple-negative BrC patients in The Cancer Genome Atlas (TCGA)-BRCA database exhibited the CE2 carcinoma ecotype. Neutrophils were more abundant in solid non-tumor tissues of BrC patients compared to their tumor tissue, displaying varying fractions across different tumor subtypes. These distinctions in cellular fractions among subtypes and ecotypes underscore the heterogeneity of the disease. The gene *LCK* has been identified as a key regulator, orchestrating neutrophil enrichment and polarization towards either pro- or anti-inflammatory states in triple-negative BrC.

Clear cell renal cell carcinoma (ccRCC) comprises 80–90% of kidney cancers globally. Over the last 15 years, significant strides have been made in targeted therapy. This progress is attributed to the advent of tyrosine kinase inhibitors, mTOR inhibitors, and immunotherapy drugs, either administered as standalone treatments or in combination with tyrosine kinase inhibitors [2]. Nevertheless, despite therapeutic progress, the rise of resistance presents a substantial challenge [3]. Small C-terminal domain phosphatases, namely CTDSP1, CTDSP2, and CTDSPL (also known as SCP1, 2, 3), play crucial roles in regulating pathways associated with carcinogenesis. Given the high mortality rate of ccRCC, there is a significant need to explore new carcinogenic mechanisms and identify pertinent tumor suppressors specific to ccRCC. In a study conducted by Krasnov et al., transfecting the Caki-1 cell line with expression constructs containing the coding regions of these genes led to the inhibition of cell growth in vitro for *CTDSP1* (*p* < 0.001) and *CTDSPL* (*p* < 0.05) but not for *CTDSP2* (contribution 3). The analysis of TCGA data revealed the differential expression of some *CTDSP* genes and their target, *RB1*. These findings were corroborated by quantitative reverse transcription polymerase chain reaction (RT-PCR) using an independent sample of primary ccRCC tumors (*n* = 52). The researchers observed *CTDSPL* downregulation and noted a positive correlation in expression for two gene pairs: *CTDSP1* and *CTDSP2* (rs = 0.76; *p* < 0.001) and *CTDSPL* and *RB1* (rs = 0.38; *p* < 0.05). Survival analysis based on TCGA data demonstrated a robust association between lower expression of *CTDSP1*, *CTDSP2*, *CTDSPL*, and *RB1* and poor survival in ccRCC patients (*p* < 0.001). Additionally, according to TCGA, *CTDSP1*, *CTDSP2*, and *RB1* exhibited differential expression in two ccRCC subtypes—ccA and ccB—with distinct survival rates. These results affirm the tumor suppressor properties of *CTDSP1* and *CTDSPL* in ccRCC, highlighting their association with the more aggressive ccRCC phenotype. Furthermore, the potential involvement of certain miRNAs in regulating phosphatase gene expression is noteworthy, but further investigation is required to elucidate this aspect [4].

Despite immunotherapy progressively establishing itself as a therapeutic standard in cancer treatment planning, only a minority of patients derive benefits, with low treatment response rates and drug resistance posing significant obstacles to the advancement of these therapies [5]. The epithelial–mesenchymal transition (EMT) process and cancer stem cells are widely recognized as pivotal factors in tumor initiation, recurrence, and metastasis, significantly influencing the effectiveness of immune checkpoint inhibitors [6]. Cuproptosis, a recently identified form of programmed cell death intricately linked to cellular metabolism, has garnered attention. Wu et al. used multiple public databases to evaluate *OLR1* expression and its relationship with prognosis in 33 cancer types and to explore correlations between *OLR1* expression levels and immune infiltration and immune checkpoint expression (contribution 4). The correlation analysis results showed that *OLR1* was positively correlated with most immune checkpoint molecules in the majority of cancer types, with a particularly strong correlation with *HAVCR2*. The Kyoto Encyclopedia of Genes and Genomes (KEGG) analysis also revealed a significant correlation between *OLR1* and the programmed death 1 (PD-1)/programmed death ligand 1 (PD-L1) pathway. To investigate the relationship between OLR1 and immunotherapy further, researchers correlated *OLR1* expression with tumor mutation burden (TMB) and microsatellite instability (MSI), which were evaluated as potential biomarkers for predicting immune-checkpoint therapy responses. Additionally, they found that *OLR1* was correlated with both TMB and MSI in specific cancer types. Moreover, tumor immune dysfunction and exclusion (TIDE) integrates the expression signatures of T-cell dysfunction and T-cell exclusion to mimic tumor immune evasion and, thus, predict clinical responses to immune checkpoint inhibitors. An elevated TIDE score correlates with an increased tendency for immune evasion and a reduced response to immune checkpoint inhibitors. Consequently, the TIDE algorithm was used to validate the relevance of *OLR1* to immunotherapy. Interestingly, the results show that HNSCC patients with high *OLR1* expression levels have higher TIDE, T-cell exclusion, and dysfunction scores but lower MSI scores. These results suggest that patients with high *OLR1* expression levels may be resistant to treatment with immune checkpoint inhibitors.

Prostate cancer (PCa) stands as the second most prevalent malignancy and the fifth-leading cause of cancer-related death among men globally, with reported figures of 1,414,259 new cases and 375,304 deaths in 2020 [1]. However, this prevalence is partly attributed to the widespread availability of prostate-specific antigen (PSA) testing, regardless of its limitations, as it is organ but not cancer-specific [7]. While locally advanced disease is potentially curable, metastatic disease presents limited therapeutic options. Targeting the androgen receptor (AR) signaling pathway remains crucial for developing novel and more effective therapies. Androgen deprivation therapy (ADT) continues to serve as the cornerstone in the management of PCa patients. The development of resistance to ADT characterizes the status of metastatic castration-resistant PCa (mCRPC), which is still linked to a dismal clinical outcome, poor prognosis, and limited therapeutic alternatives. The documented OS benefit of combining ADT with radiotherapy in localized PCa is well-established [8]. However, there is currently a lack of prospective randomized trials investigating the use of ADT in conjunction with Stereotactic Ablative Radiotherapy (SABR). It has been proposed to employ a luteinizing hormone-releasing hormone (LHRH) antagonist for a duration of 6 months, starting from day zero of SABR, particularly when treating oligometastatic disease in PCa [9]. Furthermore, PCa is characterized by its genomic instability, and 90% of mCRPC carry clinically actionable germline and somatic alterations in non-AR-related pathways [10]. DNA damage response (DDR) defects constitute a significant portion (25%) of these alterations. DDR genes play crucial roles in maintaining genomic stability, repairing DNA aberrations throughout the cell cycle, ensuring accurate mitotic cell division, and facilitating the distribution of genomic material to daughter cells. Dysfunction in DDR genes, whether inherited or acquired, results in genomic instability, an elevated mutation rate, and, consequently, heightened tumorigenesis and intra-tumor heterogeneity [11]. In recent years, the treatment landscape has shown notable improvements in the outcomes of mCRPC patients, particularly in terms of DFS and OS [12]. The second-generation AR axis-targeted agents, abiraterone acetate and enzalutamide, are currently approved as first-line treatments for asymptomatic or minimally symptomatic mCRPC patients who have not undergone prior chemotherapy. Additionally, they are approved as second-line treatments for those with symptomatic mCRPC who have progressed after docetaxel-based chemotherapy. A study by Silva J et al. aimed to explore the relationship between circulating miRNAs and treatment outcomes in patients with mCRPC treated with either abiraterone or enzalutamide, pre- or post-docetaxel therapy (contribution 5). The study’s findings identified plasmatic miR-16-5p and miR-145-5p as potential predictors of disease progression during abiraterone treatment. Additionally, plasmatic miR-20a-5p appeared to predict the OS of mCRPC patients, irrespective of the AR axis-targeted therapy employed. In silico analyses, coupled with the existing literature, highlight several targets of these miRNAs. These targets are implicated in AR-related pathways and are currently under investigation as potential therapeutic targets for mCRPC patients undergoing AR axis-targeted therapy. Considering the potential clinical benefits, there is a need for further investigation into the predictive and prognostic value of miR-16-5p, miR-145-5p, and miR-20a-5p among mCRPC patients. A comprehensive evaluation of their expression levels in PCa tissues is essential to better understand their suppressive and/or oncogenic functions. Additionally, exploring the role of these transcripts in the context of hormone sensitivity is crucial, given its implications for patient management. Despite promising results regarding miR-16-5p, miR-145-5p, and miR-20a-5p, these transcripts can target numerous mRNAs and a single mRNA can be targeted by several miRNAs. Therefore, real-world studies providing functional data on these miRNAs are imperative to comprehensively dissect the underlying biological mechanisms in mCRPC.

MiRNAs are short non-coding RNAs, typically around 22 nucleotides in length. They are highly conserved and naturally encoded in the genomes of various species, playing crucial functional roles in gene expression regulation at both transcriptional and post-transcriptional levels of their target mRNAs. This regulation extends to influencing cell function by modulating the stability and translation of mRNA across a diverse array of biological processes within cells and organisms. Consequently, miRNAs impact cell differentiation, proliferation, angiogenesis, and apoptosis [13]. These molecules can be secreted into circulation within extracellular fluids and transported to target cells through extracellular vesicles, including exosomes, microvesicles, and apoptotic bodies. This occurs under various physiological and pathological conditions, allowing miRNAs to serve as chemical messengers facilitating cell-to-cell communication [14]. Additionally, miRNAs can bind to proteins, notably Argonautes (AGO), particularly AGO2. Seyhan AA conducted a comprehensive review of dysregulated miRNA expression in cancer, with a specific focus on pancreatic cancer (contribution 6). The review highlighted the clinical applications of miRNAs as potential biomarkers that could enhance various aspects of patient care in the field of oncology, including diagnosis, prognosis, monitoring, and treatment. The widespread adoption of next-generation molecular profiling technologies, such as high-throughput next-generation RNA sequencing, including single-cell RNA sequencing, is providing researchers with more profound insights into gene dysregulation during tumorigenesis. This technological advancement has facilitated the identification of miRNAs and other non-coding RNAs for cancer screening. Additionally, these findings are instrumental in the development of RNA-targeted or RNA-based cancer therapies. Furthermore, there is a growing exploration of circulating miRNAs as potential minimally invasive biomarkers for various clinical applications, including early-stage cancer screening, subtype classification, predicting drug sensitivity for treatment strategy selection, and assessing the chemo- or radio-resistance of tumors. Moreover, miRNAs offer valuable insights into tumor evolution and the mechanisms of therapy resistance. However, numerous challenges exist in the application of circulating miRNAs as biomarkers. The widespread adoption of miRNA biomarkers in routine clinical practice faces obstacles due to concerns related to insufficient sensitivity, specificity, and selectivity, particularly in early-stage diseases and across diverse types of diseases. Therefore, there is a need for the comprehensive validation and standardization of existing miRNA profiling and analysis methodologies. This includes confirming candidate circulating miRNAs through large prospective randomized controlled trials to further assess and validate the sensitivity, specificity, selectivity, and overall applicability of potential circulating miRNA biomarkers.

Lung cancer ranks among the most prevalent cancers and stands as the primary contributor to cancer-related fatalities. The clinical characteristics of adenocarcinomas in non-smokers include a higher occurrence of pleural metastasis compared to smokers, along with variations in mutational profiles and demographics. Hamouz et al. conducted a literature review focusing on crucial signaling pathways and driver mutations (contribution 7). The tumor microenvironment (TME) in non-smokers (NS) exhibits marked differences from that of smokers. Additionally, the immune system plays a crucial role in the progression of cancer development. When non-smokers are exposed to environmental tobacco smoke (ETS), immune cells are initially mobilized to mitigate the damage caused by carcinogenic substances. *EGFR* mutations were detected in 40–60% of non-small cell lung cancer (NSCLC) patients who were non-smokers (NS), with 17% of these cases attributed to lung adenocarcinoma (LUAD). Notably, these mutations were more prevalent in those who never smoked or those who smoked rarely. However, it is crucial to note that the absence of *EGFR* mutations in smokers does not imply their non-existence; rather, it indicates that common *EGFR* mutations were more frequently observed in non-smokers, while smokers tended to exhibit a higher occurrence of uncommon single and complex rare mutations. Merely identifying the *EGFR* mutation is insufficient for predicting a patient’s response to tyrosine kinase inhibitors (TKI), as secondary *EGFR* mutations or alterations in downstream signaling pathways may be present. Therefore, thorough genotyping, particularly focusing on interactions with the *EGFR* mutation, is essential to gain a comprehensive understanding of potential signal activations in the patient. Numerous studies in NSCLC have revealed that alternative signaling pathways can contribute to resistance to EGFR-TKI. For instance, JAK2-related signaling activation, upregulating ROR1 via NKX2-1, leads to NOTCH1 overexpression, inducing EMT. Additionally, *T790M* mutation-induced EGFR-TKI resistance is associated with elevated DNA repair activity attributed to high levels of BRCA1. Furthermore, NFKB signaling has been implicated in TKI resistance for EGFR-mutant NSCLC cells, regardless of smoking history, but attempts to deactivate NFKB using a TLR-9 agonist alongside erlotinib did not significantly improve progression-free survival compared to erlotinib alone. Indeed, EGFR-TKI has demonstrated more favorable responses in LUAD patients with no smoking history, who are in the female demographic, who are of Asian ethnicity, or those with *EGFR* mutations. In NSCLC, activating mutations in the Notch signaling pathway are linked to a poorer prognosis. Notably, Notch1 is implicated in acquired resistance to EGFR-TKI in NSCLC. Furthermore, there is a positive correlation between Notch3 expression and EGFR expression, and elevated Notch3 levels are associated with an unfavorable prognosis in NSCLC. Moreover, the aberrant activation of the PI3K–AKT–mTOR pathway has been identified as a source of resistance to EGFR-TKI. This activation can occur through various mechanisms, such as the activation of tyrosine kinase receptors upstream of PI3K, PIK3CA amplifications, mutations in *KRAS*, *PI3K*, *AKT*, and *TSC1/2*, or the loss of *PTEN*. Prominent mutations leading to the activation of the PI3K–AKT–mTOR pathway include *PIK3CA* and *AKT1* mutations, as well as *PTEN* loss. Finally, genetic mutations in the PIK3CA/AKT/mTOR pathway, which represents one of the downstream pathways of EGFR, may influence the response to EGFR-TKI in NSCLC in activating *EGFR* mutations.

The primary challenge in overcoming resistance to conventional glioma treatments, such as the established Stupp protocol, lies in the pronounced heterogeneity of the TME. This microenvironment is immunosuppressive, facilitating evasion from the immune system and thereby contributing to the swift progression of the disease. Recently, there has been a growing exploration of alternative treatment approaches, including immunotherapy. Linares CA et al. conducted a review aimed at delineating the immunobiological characteristics of the glioma TME, elucidating how the TME eludes immunotherapies and highlighting ongoing efforts to navigate and counteract this intricate interplay (contribution 8). A significant portion of the tumor mass consists of immune cells, with tumor-associated myeloid cells (TAMCs) making up a substantial component. TAMCs encompass various subtypes, including tumor-associated macrophages (TAMs), myeloid-derived suppressor cells (MDSCs), dendritic cells (DCs), neutrophils, and microglia. Activated TAMs can manifest across a spectrum of phenotypes, reflecting different functional states, including the tumor-suppressive M1 or the immune-suppressive M2. The heightened accumulation of TAMs exhibiting the M2 phenotype has been associated with elevated tumor grade and diminished median OS, indicating poorer outcomes in recurrent glioblastoma. DCs are typically found in the meninges and choroid plexus but are absent in the normal brain parenchyma. In contrast, within a brain infiltrated by glioma, DCs are located within the parenchyma. The adaptive changes within the TME are heavily influenced by immune cells and the blood–brain barrier (BBB). Comprising a semipermeable membrane with endothelial cells, astrocyte foot processes, and pericytes, the BBB serves to isolate the brain from the peripheral immune system. While naïve T cells are unable to traverse the BBB, activated T cells have this capability. Consequently, the BBB meticulously controls the entry of leukocytes into the brain parenchyma, leading to diminished overall immune surveillance for gliomas in comparison to other types of tumors. While normal brain tissue does not host Treg cells, an increased abundance of these cells is observed in a brain infiltrated by glioma. This plays a pivotal role in the glioma’s ability to elude the immune system, a topic that will be further explored in subsequent sections. Chemokines, specifically CCL2 and CXCL12 secreted by glioma cells, recruit these cells to the TME. The quantity of Treg cells is associated with both the location and grade of the tumor. These cells induce compromised antigen-presenting cells (APCs), leading to a reduced capacity to activate tumor-reactive T cells. Additionally, Treg cells release factors like IL-10 and TGF-β, which inhibit the activity of other immune cells. The infiltration of M2-phenotype macrophages and Treg cells in glioblastoma further contributes to the suppression of T-cell function. Gliomas exhibit the expression of PD-L1, the primary ligand for PD-1, leading to T-cell exhaustion and anergy. Persistent antigenic stimulation within the tumor microenvironment induces T-cell exhaustion, characterized by impaired cytokine production, cytotoxicity, and proliferation. This exhaustion is mediated by immune checkpoint molecules such as PD-1 and cytotoxic T-lymphocyte-associated protein 4 (CTLA-4). Glioma immunotherapy research has primarily concentrated on four strategies: immune checkpoint inhibitors, chimeric antigen receptor T and NK cells, cancer vaccines, and oncolytic viruses. As the limitations of the TME are being addressed, combination therapies incorporating these approaches are gaining traction in the glioma context.

Molecular oncology is an expansive and continually advancing field of research. Despite the extensive study of many areas, there are still several aspects of cancer biology that remain inadequately understood. Although the papers in this issue delve into some of these areas, much remains to be discovered about the intricate biology of cancer.
